# A decade of survival in recurrent nonfunctioning oncocytic adrenocortical carcinoma in a young Filipina

**DOI:** 10.1210/jcemcr/luag254

**Published:** 2026-09-16

**Authors:** Ranee Joy Jacobo, Quennie Fae de Leon, Patrick Wilson Lim, Fidela Anne Badilles, Patricia Maningat

**Affiliations:** Center for Diabetes, Thyroid, and Endocrine Disorders, St. Luke's Medical Center, Global City 1634, Philippines; Center for Diabetes, Thyroid, and Endocrine Disorders, St. Luke's Medical Center, Global City 1634, Philippines; Center for Diabetes, Thyroid, and Endocrine Disorders, St. Luke's Medical Center, Global City 1634, Philippines; Center for Diabetes, Thyroid, and Endocrine Disorders, St. Luke's Medical Center, Global City 1634, Philippines; Center for Diabetes, Thyroid, and Endocrine Disorders, St. Luke's Medical Center, Global City 1634, Philippines

**Keywords:** oncocytic adrenocortical carcinoma, mitotane, long-term survival

## Abstract

Adrenocortical carcinoma (ACC) is a rare endocrine malignancy with high recurrence risk and limited long-term survival, particularly after metastatic spread. Oncocytic ACC is an uncommon variant that may behave more indolently but can still recur. We report a 30-year-old Filipina with a nonfunctioning oncocytic ACC initially treated with laparoscopic adrenalectomy (R1 margin; Ki-67 10%-20%). She developed local recurrence at 2.5 years, underwent open resection (R1; Ki-67 20%) followed by adjuvant tumor-bed radiotherapy and mitotane, which was discontinued because of intolerance and cost. After a lapse in follow-up, she developed a solitary hepatic metastasis resected with negative margins; planned etoposide, doxorubicin, and cisplatin chemotherapy was stopped after 3 cycles because of ototoxicity. Subsequent surveillance identified a paraumbilical nodal/subcutaneous metastasis resected with negative margins. More than 10 years from diagnosis, she remains under active surveillance without radiographic evidence of active disease. This case highlights the potential for prolonged survival in selected patients with recurrent/metastatic ACC through iterative multidisciplinary decision-making, medical management, and repeat metastasectomy when feasible.

## Introduction

Adrenocortical carcinoma (ACC) has an incidence of approximately 1 to 2 cases per million annually and is characterized by frequent recurrence even after surgery [1]. Contemporary guidelines emphasize complete resection when feasible, risk-adapted adjuvant therapy (particularly for high-risk features such as R1 margins and higher Ki-67), and long-term surveillance [2]. The etoposide, doxorubicin, and cisplatin (EDP) plus mitotane regimen is the best-supported systemic option for advanced ACC, improving progression-free survival compared with streptozocin plus mitotane, though overall survival remains limited in trial populations [1]. Oncocytic adrenal tumors require specific diagnostic criteria such as the Lin-Weiss-Bisceglia system to define malignancy [3]. Although oncocytic adrenocortical carcinoma (OAC) generally exhibits a more indolent clinical course and a more favorable prognosis than conventional ACC, the presence of distant metastases remains a major adverse prognostic factor, similar to conventional ACC [4]. Long-term survival beyond 10 years remains uncommon, especially after metastatic disease, making this patient's clinical course instructive.

## Case presentation

**Figure 1:**
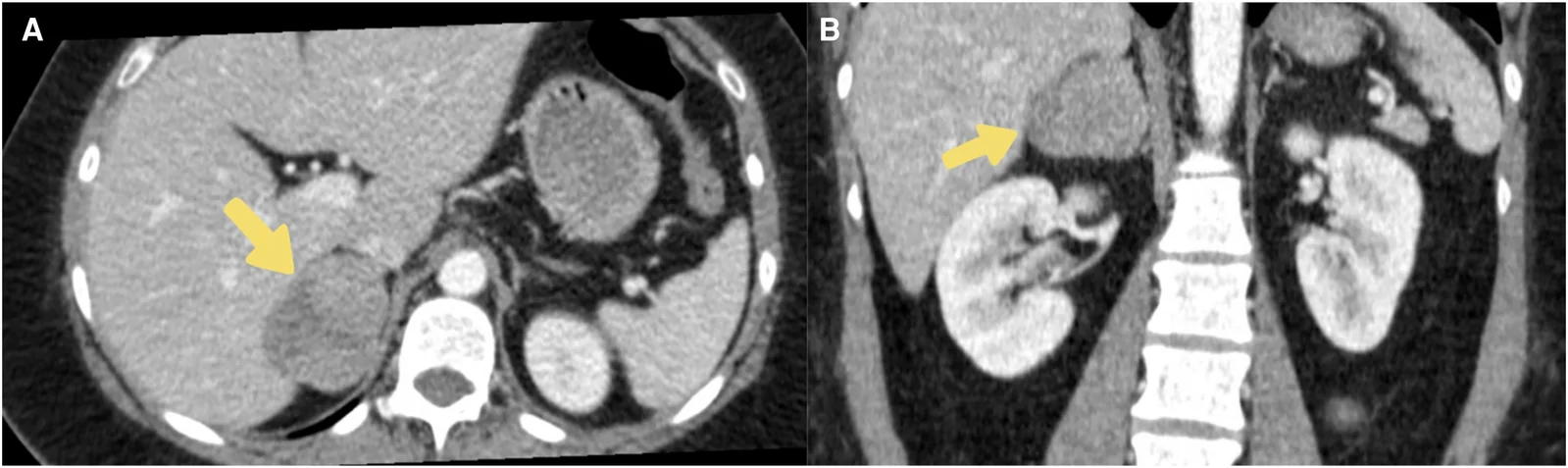
Contrast-enhanced computed tomography (CT) urogram demonstrating a right adrenal mass. A (left, axial view) and B (right, coronal view) show a large, well-circumscribed, heterogeneous mass arising from the medial limb of the right adrenal gland, measuring approximately 5.2 × 4.8 × 4.0 cm, with heterogeneous enhancement (yellow arrows). The mass abuts the inferomedial aspect of the liver. The left adrenal gland appears unremarkable.

A 30-year-old Filipina presented in April 2015 with weight gain, new-onset hypertension, intermittent headaches, occasional palpitations, and dull right flank pain. She had no cushingoid features, virilization, or classic paroxysmal spells. Her history included bronchial asthma, polycystic ovarian syndrome, prediabetes, and dyslipidemia. Family history was notable for metabolic and thyroid conditions without known endocrine tumor syndromes. She was a former smoker, occasional alcohol drinker, and works as a teacher. An abdominal ultrasound detected an incidental right adrenal mass. A contrast-enhanced computed tomography (CT) urogram, requested by the attending urologist, showed a large, heterogeneous right adrenal lesion (∼5 cm), Fig. 1. Upon admission, the patient was referred to endocrinology for clearance before planned laparoscopic surgery.

## Diagnostic assessment

Biochemical workup supported a nonfunctioning adrenal mass: aldosterone-renin ratio and potassium were normal; 1-mg overnight dexamethasone suppression demonstrated appropriate cortisol suppression; 24-hour urinary fractionated metanephrines, serum dehydroepiandrosterone sulfate and total testosterone were within normal limits. A baseline 18F-fluorodeoxyglucose positron emission tomography/computed tomography (18F-FDG PET/CT) scan was not requested at this time but may have improved initial staging by identifying occult metastatic disease.

In May 2015, she underwent right laparoscopic adrenalectomy. Histopathology revealed OAC with malignant features consistent with Lin-Weiss-Bisceglia criteria. Ki-67 index was 10% to 20%, and tumor was present at the inked margin (R1). Immunohistochemistry supported adrenocortical origin (calretinin, inhibin, Melan-A positive). This case was staged as European Network for the Study of Adrenal Tumors stage II at initial diagnosis, although a more extensive imaging procedure like a PET-CT scan was not done.

**Figure 2:**
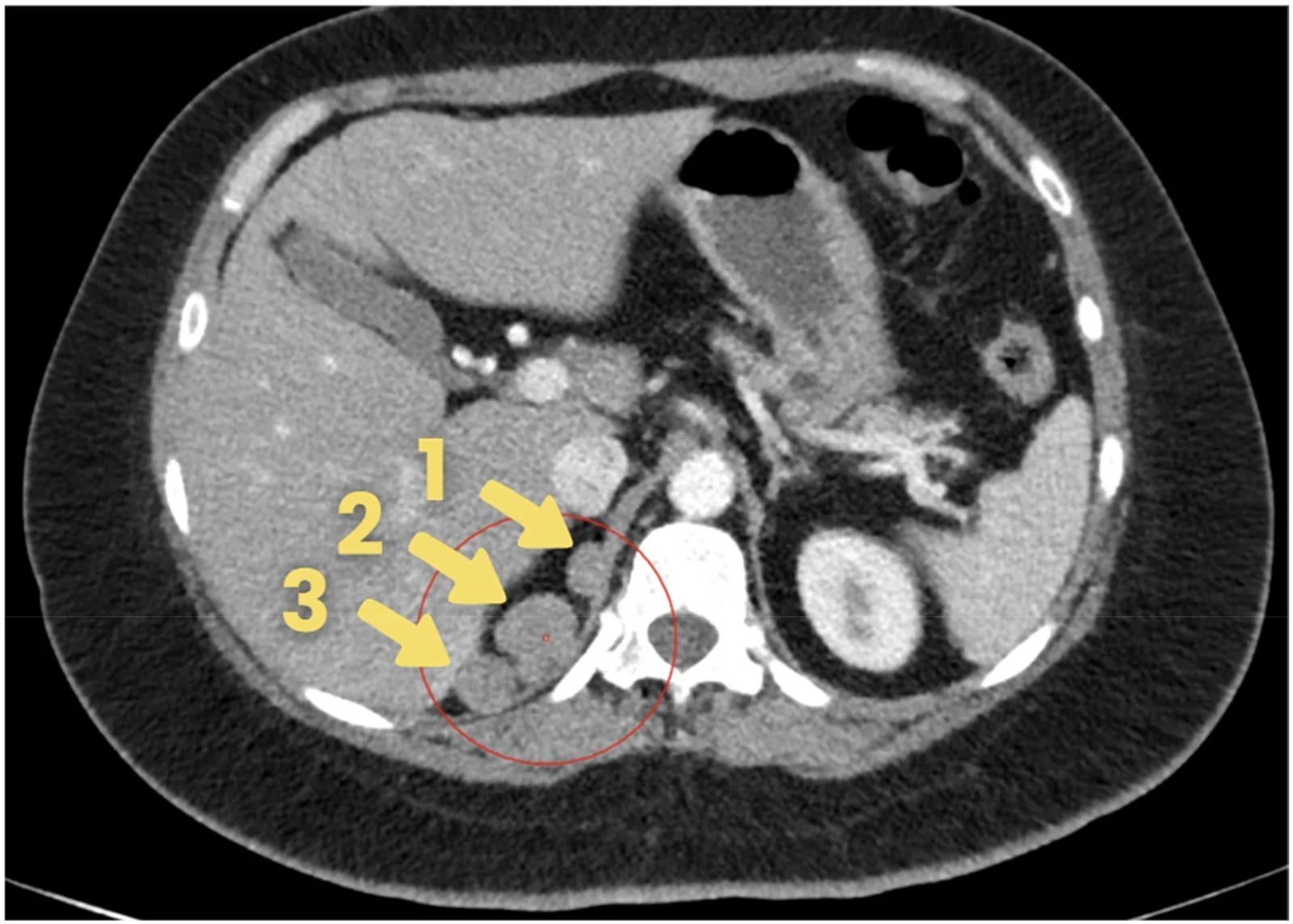
Contrast-enhanced computed tomography (CT) scan of the abdomen showing suspected recurrent right adrenal lesions. Axial image demonstrates at least 3 lobulated, mildly enhancing ovoid lesions in the right suprarenal region (arrows and circled area), measuring 0.9 to 2.0 cm, suspicious for recurrence. Quantitative assessment shows: (1) 49 Hounsfield unit (HU) with 26% washout, (2) 39 HU with 37% washout, and (3) 36 HU with 23% washout.

Surveillance whole-abdomen CT scan initially showed no residual or metastatic disease. In November 2017, CT demonstrated 3 lobulated enhancing lesions (0.9-2.0 cm) in the right suprarenal bed, suspicious for recurrence, Fig. 2. Biochemical testing remained unremarkable. In January 2018, open resection of recurrent tumor was performed; histopathology confirmed recurrent ACC with positive margins (R1) and Ki-67 ∼20%. External review at the Mayo Clinic USA corroborated malignant ACC and margin positivity.

**Figure 3:**
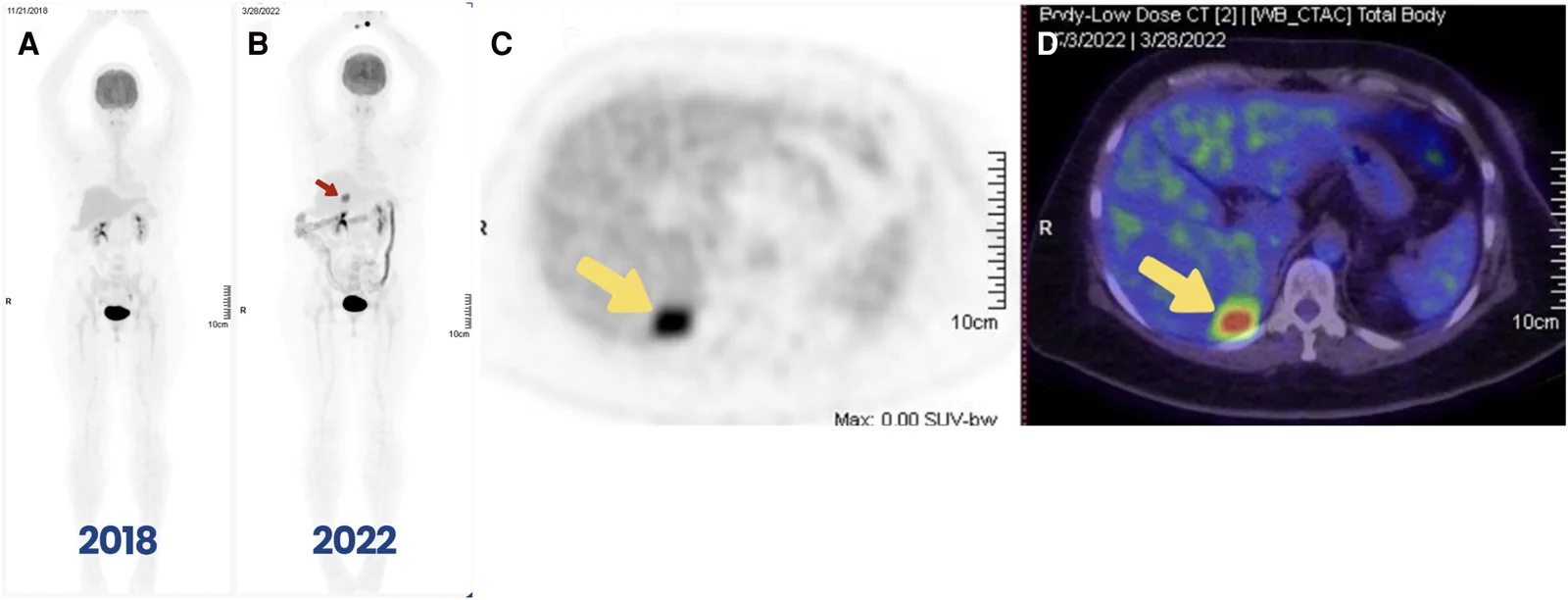
18F-fluorodeoxyglucose positron emission tomography/computed tomography (18F-FDG PET/CT) demonstrating a hypermetabolic hepatic lesion consistent with recurrence. A (left) and B (second from left) show maximum intensity projection images from 2018 and March 2022, respectively, demonstrating interval development of a focal hypermetabolic lesion in the right upper abdomen (red arrow). C (third image, axial PET) and D (right, fused PET/CT) localize this lesion to hepatic segment VII (yellow arrows), measuring approximately 2.0 × 2.5 cm with a maximum standardized uptake value (SUV max) of 10.1, suspicious for recurrent disease.

**Figure 4:**
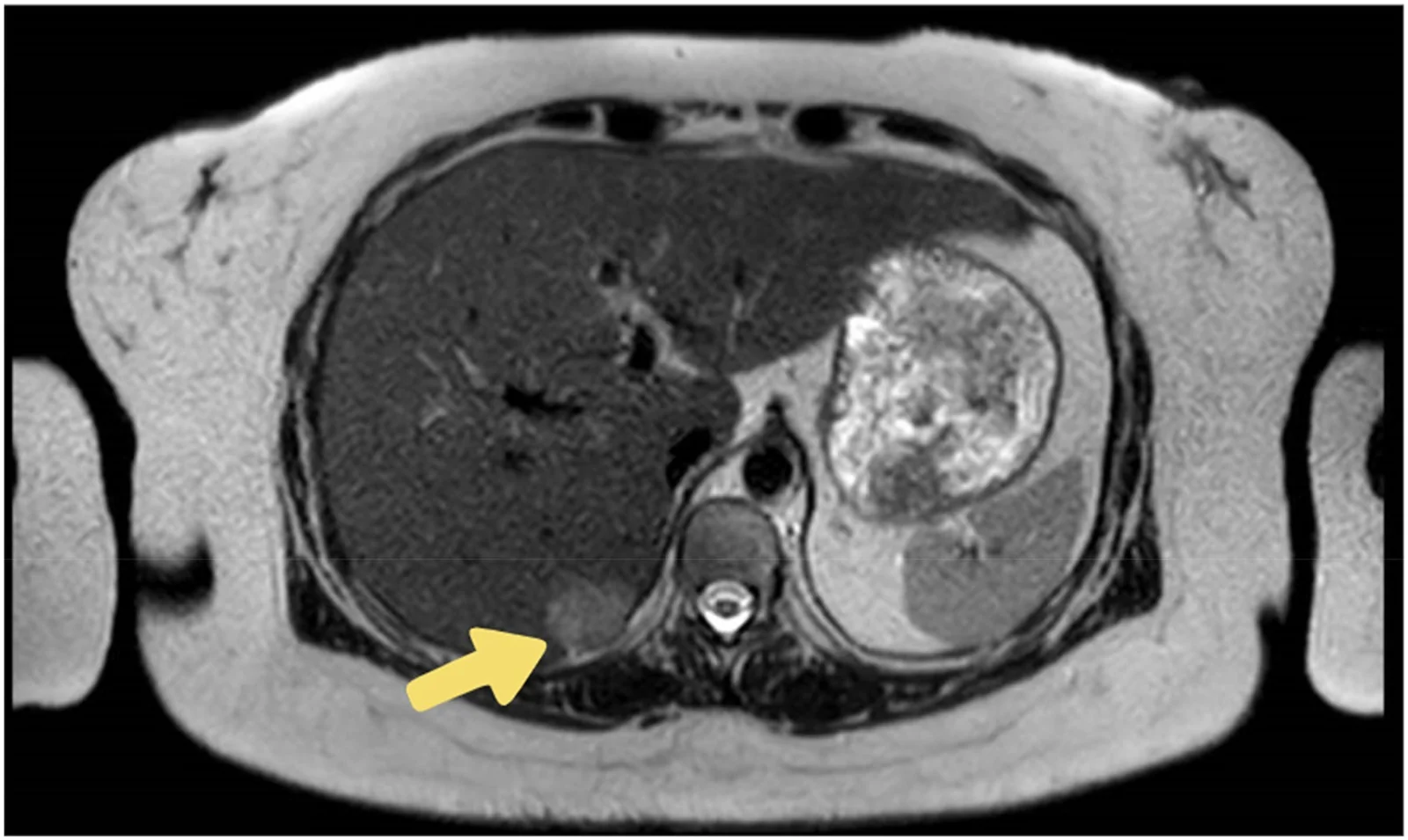
Magnetic resonance imaging (MRI) of the liver demonstrating metastatic lesion in segment VII. Axial image shows a right hepatic lobe mass in segment VII (yellow arrow), measuring approximately 2.2 × 3.2 × 3.1 cm (previously 2.0 × 2.5 cm), with early irregular enhancement and absence of contrast uptake on the hepatobiliary phase, findings suggestive of metastasis in a patient with known adrenocortical carcinoma.

After a period of surveillance, 18F-FDG PET/CT in March 2022 showed a hypermetabolic hepatic segment VII lesion, Fig. 3; and magnetic resonance imaging supported metastatic disease, Fig. 4. In May 2022, hepatic segmentectomy confirmed metastatic ACC with negative margins.

**Figure 5:**
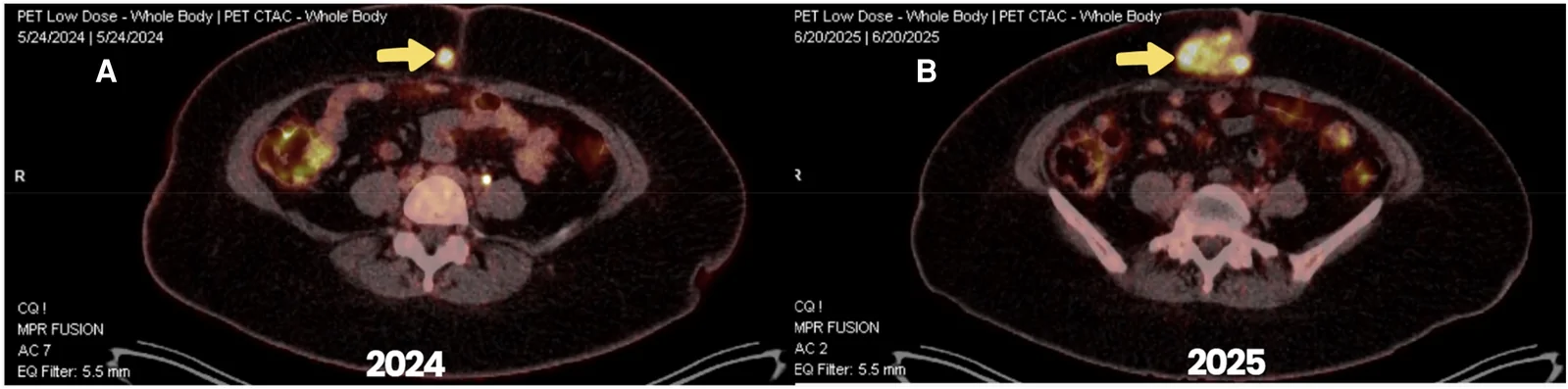
18F-fluorodeoxyglucose positron emission tomography/computed tomography (18F-FDG PET/CT) demonstrating progression of a hypermetabolic subcutaneous lesion. A (left) and B (right) show axial fused PET/CT images from May 2024 and June 2025, respectively, demonstrating an increase in size and metabolic activity of a hypermetabolic subcutaneous lesion/lymph node in the right paraumbilical region (arrows), consistent with progressive metastatic disease.

Whole-body 18F-FDG PET/CT in June 2023 showed no hypermetabolic recurrence. Annual imaging in May 2024 noted a hypermetabolic right paraumbilical node/lesion that enlarged by June 2025, prompting excision, Fig. 5. Histopathology again showed metastatic ACC with negative margins.

## Treatment

2015 (primary disease): Right laparoscopic adrenalectomy. Despite R1 resection and Ki-67 10% to 20% (high-risk features), initial postoperative strategy after multidisciplinary discussion was for close surveillance rather than immediate mitotane treatment, reflecting uncertainty at the time regarding benefit in oncocytic variants and evolving evidence in adjuvant settings.

2017-2018 (first local recurrence): Following CT-detected right adrenal bed recurrence, open resection was performed (January 2018). Histology showed recurrent ACC with R1 margins and a persistently high Ki-67 (∼20%). She then received adjuvant tumor-bed radiotherapy (28 fractions) and mitotane. Mitotane levels remained subtherapeutic as the patient only tolerated a dose of 3 grams/day because of adverse effects. Glucocorticoids (hydrocortisone, later shifted to prednisone) was given during mitotane treatment. Mitotane was eventually discontinued in November 2019 because of cost.

2022 (solitary hepatic metastasis): Hepatic segmentectomy was performed with negative margins. Because advanced ACC often warrants systemic therapy and EDP-mitotane has demonstrated improved progression-free survival, she proceeded with EDP chemotherapy but without mitotane because of prior intolerance. The planned 6 cycles were discontinued after 3 cycles because of ototoxicity.

2025 (paraumbilical nodal/subcutaneous metastasis): Excision achieved negative margins. Given low-volume disease and prior systemic therapy intolerance, mitotane was reintroduced cautiously at a low dose, aligning with evidence that a subset of advanced ACC patients, particularly those with lower tumor burden and delayed recurrence, may achieve prolonged disease control on mitotane monotherapy [5]. Glucocorticoids were resumed during mitotane treatment.

## Outcome and follow-up

Our patient has survived more than 10 years from initial diagnosis of OAC despite multiple recurrences and metastases. Disease control has been achieved through repeated complete resections, radiotherapy after local recurrence, and intermittent systemic therapy limited by toxicity. Following the most recent (2025) resection, surveillance has consisted of whole-body 18F-FDG PET-CT together with biochemical evaluation (including morning cortisol, aldosterone, renin, dehydroepiandrosterone sulfate, total testosterone) every 6 to 12 months to monitor for recurrent functional or structural disease.

The patient shared the following reflections and we share some excerpts on her long-term experience living with recurrent OAC:

“Living with ACC has been physically and emotionally challenging. While surgeries, radiation, and chemotherapy have controlled my disease, the fear of recurrence and the anxiety of waiting for scan results never completely disappear. The treatments have left me fatigued, and I have learned to adjust my daily life and prioritize rest. I hope health care providers recognize that patients with rare cancers need not only effective treatment but also empathy, reassurance, and emotional support throughout their journey. To others living with ACC, it is okay to feel scared or tired. Coping is not only about fighting the disease but also about caring for yourself.”

## Discussion

This case highlights that prolonged survival in metastatic ACC, although uncommon, may be achievable in select patients through aggressive local control and repeated resection. Notably, this tumor represents the oncocytic variant of ACC, a rare subtype characterized by a predominance of oncocytic cells. Because the conventional Weiss criteria may overestimate malignancy in oncocytic adrenal tumors because of their inherent cytologic features, the Lin-Weiss-Bisceglia system is the preferred classification system, defining malignancy by the presence of at least 1 major criterion (mitotic rate >5/50 high-power fields, atypical mitoses, or venous invasion) [3]. Current literature suggests that OAC generally has a more favorable prognosis than conventional ACC, with longer overall survival and often a more indolent course. However, oncocytic histology should not be interpreted as biologically benign. Prognosis remains heterogeneous and is influenced by the adverse factors recognized in conventional ACC, including incomplete resection, elevated Ki-67 index, advanced European Network for the Study of Adrenal Tumors stage, and metastatic disease [4-6]. Thus, patients with OAC should be approached according to individualized risk rather than histologic subtype alone.

Although the patient initially presented with hypertension and weight gain, extensive endocrine evaluation demonstrated a nonfunctioning tumor. These clinical features were therefore considered more likely attributable to preexisting metabolic syndrome, obesity, and recently diagnosed hypertension rather than hormonal hypersecretion from the adrenal neoplasm.

Several factors may explain this patient's prolonged survival despite metastatic disease. First, her recurrences were oligometastatic and appeared after relatively long disease-free intervals, allowing potentially curative local treatment rather than purely palliative systemic therapy. Second, each recurrence was actively investigated and treated with complete surgical resection when feasible, including resection of adrenal bed recurrence, hepatic metastasectomy, and excision of the paraumbilical metastatic lesion. Third, close surveillance enabled early detection of low-volume recurrence. Finally, care was continuously individualized through a multidisciplinary team, balancing oncologic benefit against treatment-related toxicity from mitotane and chemotherapy.

Surgery remains the cornerstone of management. Even after recurrence, reoperation and metastasectomy can confer meaningful survival benefit in selected patients, especially with limited metastatic burden, longer disease-free intervals, and complete resectability. Systematic reviews and pooled analyses support reoperation in recurrent ACC, including in some patients undergoing debulking, though complete resection is consistently associated with better outcomes [7].

Adjuvant therapy decisions are complex and risk-adapted. The ADIUVO trial evaluated adjuvant mitotane in low-grade, localized ACC after complete resection and did not demonstrate clear benefit, reinforcing that adjuvant mitotane is not uniformly indicated for low-risk disease. However, our patient's R1 margins and higher Ki-67 placed her outside that low-risk population and supported considering adjuvant therapy at recurrence [8]. Modern guidelines recommend mitotane for high-risk patients and acknowledge tumor-bed radiotherapy as an option to reduce local recurrence risk [2]. For OAC, treatment principles are largely extrapolated from conventional ACC because prospective subtype-specific trials are lacking. Therefore, management should be guided by stage, margin status, Ki-67 index, recurrence pattern, tumor burden, and patient tolerance rather than by oncocytic histology alone.

Systemic therapy is often constrained by toxicity. The FIRM-ACT trial established EDP plus mitotane as a preferred first-line regimen in advanced ACC based on improved progression-free survival [1]. In practice, mitotane-related adverse effects (and the need for therapeutic drug monitoring and glucocorticoid replacement) and chemotherapy toxicity can limit treatment intensity or duration. In resource-limited settings such as the Philippines, financial barriers may further limit the access to prolonged mitotane therapy. This patient's adverse effects, cost, and inability to maintain mitotane and EDP highlight the need for individualized strategies, supportive care, and realistic shared decision-making. Nevertheless, long-term survival was obtained with repeated complete resection and multidisciplinary management.

Emerging systemic options may be considered when standard therapies fail or are not tolerated. Pembrolizumab has demonstrated activity in advanced ACC, with responses observed even without clear predictive value from microsatellite instability (MSI) or programmed death-ligand 1 expression in some cohorts, making immunotherapy a potential option in later lines despite low programmed death-ligand 1 Combined Positive Score in this case [9]. Cabozantinib has also shown encouraging activity in a phase II study in advanced ACC [10]. Although not used in this patient to date, these represent plausible future considerations should recurrence occur.

Finally, molecular profiling did not reveal actionable targets in this case, but it remains relevant for trial eligibility and for identifying rare actionable alterations as the therapeutic landscape evolves.

In summary, this case demonstrates that although OAC generally has a more favorable prognosis than conventional ACC, high-risk pathological features can lead to recurrent metastatic disease. Nevertheless, prolonged survival may be achieved through early detection of recurrence, repeated complete resection, individualized therapy, coordinated multidisciplinary care, and comprehensive survivorship care, including early psychological or psychiatric support when appropriate and encouragement to build a strong social support network.

## Learning points

ACC has a high recurrence risk even after initial resection; long-term structured surveillance is essential.

- OAC may still recur and metastasize, particularly with high-risk features such as R1 margins and elevated Ki-67.
- Selected patients with limited recurrent/metastatic disease may achieve prolonged survival through repeated complete resections and multidisciplinary care.
- Systemic therapies for advanced ACC, including mitotane, cytotoxic chemotherapy, immunotherapy, and targeted multikinase inhibitors, are often limited by toxicity and require careful multidisciplinary management.

## Data Availability

Original data generated and analyzed during this study are included in this published article.

## Abbreviations

18F-FDG PET/CT: 18F-fluorodeoxyglucose positron emission tomography/computed tomography
ACC: adrenocortical carcinoma
CT: computed tomography
EDP: etoposide doxorubicin and cisplatin
OAC: oncocytic adrenocortical carcinoma
